# Interrater agreement between children’s self-reported and their mothers’ proxy-reported dental anxiety: a Chinese cross-sectional study

**DOI:** 10.1186/s12903-023-02834-1

**Published:** 2023-03-10

**Authors:** Su-Wei Fu, Shen Li, Zhi-Yan Shi, Qing-Li He

**Affiliations:** 1 Department of Stomatology, Henan Provincial People’s Hospital, People’s Hospital of Zhengzhou University, Zhengzhou, Henan Province China; 2 Graduate School, University of Perpetual Help System Dalta, Las Piñas, Philippines

**Keywords:** Dental anxiety, Factor, Interrater agreement, Parents, Proxy-reported, Children, Self-reported

## Abstract

**Background:**

Children's dental anxiety is common in dental clinics. This study aimed to determine the interrater agreement between children’s self-reported and their mothers’ proxy-reported dental anxiety and its affecting factors.

**Methods:**

In this cross-sectional study performed in a dental clinic, primary school students and their mothers were assessed for enrollment eligibility. The Modified Dental Anxiety Scale plus Facial Image Scale (MDAS-FIS) was employed to test both the children’s self-reported and their mothers’ proxy-reported dental anxiety independently. The interrater agreement was analyzed using percentage agreement and the linear weighted kappa (k) coefficient. Factors affecting children’s dental anxiety were analyzed using univariate and multivariate logistic regression models.

**Results:**

One hundred children and their mothers were enrolled. The median ages of the children and mothers were 8.5 and 40.0 years old, respectively, and 38.0% (38/100) of the children were female. The scores of children’s self-reported dental anxiety were significantly higher than their mothers' proxy-reported dental anxiety (MDAS-Questions 1–5, all *p* < 0.05); moreover, there was no agreement between the two groups in terms of all anxiety hierarchies (kappa coefficient = 0.028, *p* = 0.593). In the univariate model, a total of seven factors (age, gender, maternal anxiety, number of dental visits, mother’s presence or absence, oral health status, and having siblings or not) were involved for analysis, and age [every 1-year increase, odds ratio (OR) = 0.661, 95% confidence interval (CI) = 0.514–0.850, *p* = 0.001], several dental visits (every 1 visit increase, OR = 0.409, 95% CI = 0.190–0.880, *p* = 0.022), and mother presence (OR = 0.286, 95% CI = 0.114–0.714, *p* = 0.007) were affecting factors. In the multivariate model, only age (every 1 year increase) and maternal presence were associated with 0.697-fold (95% CI = 0.535–0.908, *p* = 0.007) and 0.362-fold (95% CI = 0.135–0.967, *p* = 0.043) decreases in the risk of children’s dental anxiety during dental visits and treatment, respectively.

**Conclusion:**

There was no significant agreement between elementary school students’ self-reported dental anxiety and mothers’ proxy ratings of children’s dental anxiety, which suggests that self-reported dental anxiety by children should be encouraged and adopted, and the mother’s presence during dental visits is strongly recommended.

## Introduction

Dental anxiety and phobia are known barriers to receive regular dental care in many anxious patients [1]. Dental anxiety or fear is common among children seeking dental treatment [2–4]. Severe dental anxiety and fear in children attending dental clinics will not only lead to the failure of the normal process of dental treatment but also lay a psychological shadow for adult dental anxiety and fear [5–7]. Timely detection and appropriate intervention can shorten the time and frequency of treatment and help relieve the economic and psychological burden of patients because prolonged or multiple services require more time or cost and, importantly, may result in painful memories for the children and parents. Therefore, how identifying patients' dental anxiety or fear in advance, especially in children, determining its degree, and analyzing the factors affecting dental anxiety are particularly important.

Questionnaire-based assessment of dental anxiety is the most commonly used method of dental anxiety assessment in pediatric patients [5]. However, given that many children cannot express their dental anxiety clearly and that nearly all children require parents to visit the dental clinic, there are at least two types of dental anxiety for elementary school students who are not very expressive, i.e., children’s self-reported and parents’ reported dental anxiety. Meanwhile, there are few international studies on whether there is a difference between children's self-reported and their parents’ proxy-reported dental anxiety [1]. In the process of dental visits, parents of children with dental anxiety may underestimate or overestimate the state of children with dental anxiety, so it may harm the process of dental visits and the mental health of children [1, 8], especially for underestimation risk, given that a consequence of the underestimation is the risk of overlooking some children’s needs for special attention, and therefore, they will suffer from a higher degree of dental fear [8]. Therefore, identifying which types of dental anxiety can reflect the true dental anxiety of children themselves is important. Additionally, analyzing the influencing factors of true dental anxiety (self-reported or parent-reported) is reasonable because carrying out targeted interventions based on these factors is beneficial to the management of dental anxiety in children and even their oral and mental health in their adult life. In the current study, we first investigated the interrater agreement between children’s self-reported and their mothers’ proxy-reported dental anxiety and then explored the potential factors of children’s dental anxiety.

## Methods

### Study population

In this cross-sectional study performed in the Department of Stomatology in Henan Provincial People's Hospital, China, primary school students and their mothers were potentially enrolled. The inclusion criteria were as follows: (1) the child studied in elementary school and aged 6–12 years; (2) the child and his or her parents living in Zhengzhou City (provincial capital) of Henan Province; (3) the child and his or her mother who were willing to take the survey; and (4) child who needed dental treatment. The exclusion criteria were as follows: (1) highly uncooperative child or mother; (2) child having any kind of systemic disease; (3) child taking regular medications; and (4) the child or his or her parents were cognitively impaired or doubt about the study.

### Research instrument

The Modified Dental Anxiety Scale (MDAS) plus Facial Image Scale (FIS) [9, 10], i.e., MDAS-FIS, was used to test dental anxiety for both children and their mothers in this study. The MDAS comprises five standard questions (items) to evaluate the anxiety levels of children ranging from “Not anxious” to “Extremely Anxious” (Table 1) [9]. This is one of the most reliable methods to measure dental anxiety. Most importantly, the MDAS is suitable for both children and adults, which could maintain the consistency of the assessment system for both of them. Furthermore, to easily respond to the question by the participants, the FIS is involved in evaluating anxiety levels by making the children or mothers choose a particular facial expression [10]. The facial expressions ranging from a score of 1–5 are the same as the “Not Anxious” to “Extremely Anxious” of MDAS. Therefore, a new scale was developed by combining the MDAS and FIS and named MDAS-FIS. The total score is a sum of all five items, range 5–25: the cut-off is 19 or above (i.e., ≥ 19 and < 19 indicate having dental anxiety or not having), which indicates a highly dentally anxious participant, possibly dentally phobic. Additionally, before the performance of this study, we performed a pilot study using several other scales to test dental anxiety, and we found that combining the MDAS and FIS (MDAS-FIS) is most acceptable for both children and their mothers.

**Table 1:** Modified version of the Dental Anxiety Scale (MDAS)

1	If you had to go to the dentist tomorrow for a check-up, how would you feel about it?
(a)	I would look forward to it as a reasonably enjoyable experience
(b)	I would not care one way or the other
(c)	I would be a little uneasy about it
(d)	I would be afraid that it would be unpleasant and painful
(e)	I would be very frightened of what the dentist would do
2	When you are waiting in the dentist's office for your turn in the chair, how do you feel?
(a)	Relaxed
(b)	A little uneasy
(c)	Tense
(d)	Anxious
(e)	So anxious that I sometimes break out in a sweat or almost feel physically sick
3	When you are in the dentist's chair waiting while the dentist gets the drill ready to begin working on your teeth, how do you feel?
(a)	Relaxed
(b)	A little uneasy
(c)	Tense
(d)	Anxious
(e)	So anxious that I sometimes break out in a sweat or almost feel physically sick
4	Imagine you are in the dentist's chair to have your teeth cleaned. While you are waiting and the dentist or hygienist is getting out the instruments which will be used to scrape your teeth around the gums, how do you feel?
(a)	Relaxed
(b)	A little uneasy
(c)	Tense
(d)	Anxious
(e)	So anxious that I sometimes break out in a sweat or almost feel physically sick
5	If you were about to have a local anesthetic injection in your gum, above an upper back tooth, how would you feel?
(a)	Relaxed
(b)	A little uneasy
(c)	Tense
(d)	Anxious
(e)	So anxious that I sometimes break out in a sweat or almost feel physically sick

a = 1, b = 2, c = 3, d = 4, e = 5. Total possible = 20. Anxiety rating: 9–12 = moderate anxiety but have specific stressors that should be discussed and managed; 13–14 = high anxiety; 15–20 = severe anxiety (or phobia), may be manageable with the Dental Concerns Assessment but might require the help of a mental health therapist

### Data collection

The dental anxiety of the children and their mothers was tested independently while waiting for the appointment (treatment) in a quiet and relaxing waiting room; therefore, the enrolled children and their mothers did not have to be dentally anxious to take part in the study or survey itself. Meanwhile, the following potential factors affecting child dental anxiety are collected before treatment: (1) Age; (2) Gender; (3) Maternal anxiety; (4) Number of dental visits; (5) Mother’s presence or absence; (6) Oral health status (whether to brush teeth at least once a day); and (7) Having siblings or not.

### Sample size estimation

Considering the results of previous studies, there is a more than 5% difference between children's self-reported and parents’ proxy-reported dental anxiety [1], and the number of participants needed to capture the difference that occurred in > 5% of participants was calculated to be 92 with a significance level of 0.05. In addition, according to the relevant law and regulations from the State Food and Drug Administration of China, the probability of observing 5% of the differences between the two groups is greater than 99% after completing the observation of 100 patients in each group [11].

### Statistical analysis

Continuous variables are summarized as either the means ± standard deviations or the medians and ranges, as appropriate. The percentage of patients in each category was calculated for categorical variables. The percentages were compared between the two groups using the chi-square test. A kappa consistency test was used to determine the interrater agreement of the scores and hierarchies of dental anxiety reported by the children themselves and their mothers. The grading standards of kappa coefficients are as follows: 0.0–0.20 is a very low consistency, 0.21–0.40 is general consistency, 0.41–0.60 is a medium consistency, 0.61–0.80 is a high consistency, and 0.81–1.0 is almost complete consistency. Univariate and multivariate logistic regression models were used for the statistical analysis of factors affecting elementary school students’ dental anxiety. All statistical levels were set at 0.05. The analyses were performed using SPSS software 25.0 for Windows (SPSS Inc. Chicago, IL, USA).

## Results

### Demographic characteristics of enrolled children and mothers

**Fig. 1:**
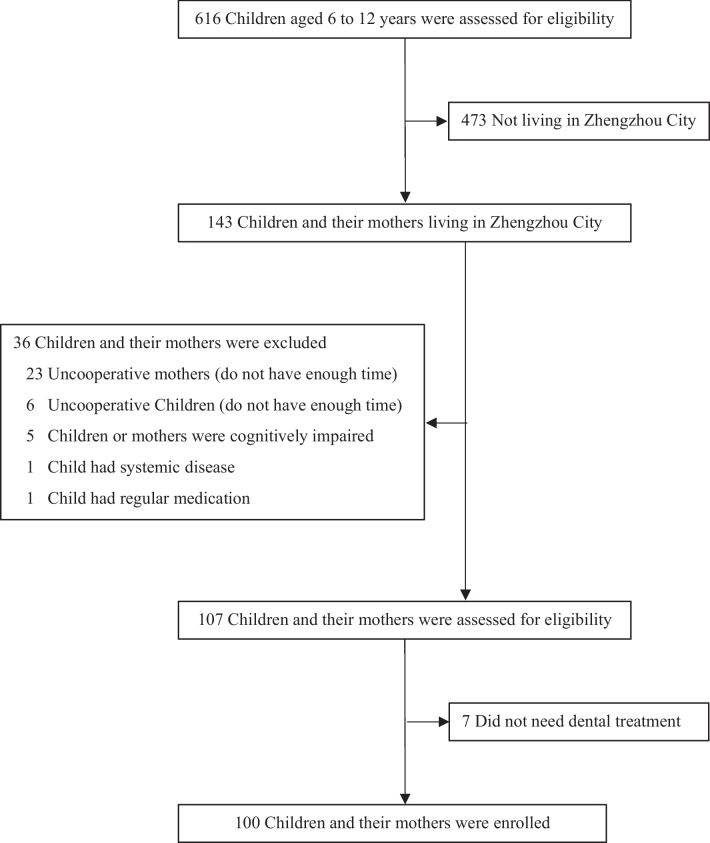
Participant enrollment

From January 2021 to May 2022, a total of 616 children and their mothers were assessed for eligibility, and 100 primary school students and 100 of their mothers living in Zhengzhou City of Henan Province were enrolled (Fig. 1). The median ages of the enrolled children and mothers were 8.5 (7.0–10.8) and 40.0 (34.0–42.0) years old, respectively, and 38% (38/100) of the children were female. A total of 26 (26%) children were grade 1 students, 63 (63%) of whom had a history of dental visits, and 66 (66%) of whom had siblings. Other characteristics are presented in Table 2.

**Table 2:** Demographic characteristics of participants

Parameters	Children (n = 100)	Mothers (n = 100)
Age, years	8.5 (7.0–10.8)	40.0 (34.0–42.0)
Sex, female	38 (38)	100 (100)
Grade 1	26 (26)	–
Grade 2	24 (24)	–
Grade 3	13 (13)	–
Grade 4	12 (12)	–
Grade 5	12 (12)	–
Grade 6	13 (13)	–
Dental visits		–
Not having dental visit previously	63 (63)	–
Having dental visit previously	37 (37)	–
Mean number	0.6 ± 0.9	–
One visit	24 (34)	–
Two visits	9 (9)	–
Three visits	3 (3)	–
Four visits	1 (1)	–
Sibling		–
Not having	66 (66)	
Having one	44 (44)	
Mother’s education background		
Bachelor’s degree	–	63 (63)
Master’s degree	–	31 (35)
Doctorate degree	–	6 (6)

Data are presented as the median (interquartile range), mean ± standard deviation, or n (%)

### The scores of children’s self-reported and mothers’ proxy-reported dental anxiety

The scores of dental anxiety reported by mothers were lower than those reported by their children, and a total of 30 and 6 children and mothers had total scores of more than 19, respectively (Table 3), which indicates that the participants were highly dentally anxious and possibly dentally phobic.

**Table 3:** Dental anxiety scores reported by children and their mothers

Parameters	Children’ self-reported scores	Parents’ proxy-reported scores
MDAS-question 1	3.1 ± 1.2	2.7 ± 1.2
MDAS-question 2	3.4 ± 1.2	2.7 ± 1.3
MDAS-question 3	3.8 ± 1.1	3.3 ± 1.1
MDAS-question 4	3.5 ± 1.2	2.8 ± 1.2
MDAS-question 5	3.7 ± 1.1	3.5 ± 1.1
MDAS-total	17.4 ± 2.3	14.8 ± 2.4
Total ≥ 19, n (%)	30 (30)	6 (6)

*MDAS* Modified version of dental anxiety scale

### The interrater agreement of the children’s self-reported and mothers’ proxy-reported dental anxiety

The kappa consistency test found that MDAS-Question 5 (kappa = 0.425, *p* < 0.001) had the highest consistency between children’s self-reported and mothers’ proxy-reported dental anxiety, followed by MDAS-Question 3 (kappa = 0.230, *p* < 0.001). It is worth noting that, even for MDAS question 5 with the highest consistency, children's self-reported dental anxiety was still underestimated by mothers' proxy-reported dental anxiety by 43% (Table 4).

**Table 4:** Consistency analysis of children's self-reported and their mothers' proxy-reported dental anxiety scores

Parameters	Consistency, n (%)	Kappa coefficient (95% CI)	*p* value
MDAS-question 1	32 (32)	0.143 (0.031–0.255)	0.003
1	7 (7)	–	–
2	5 (5)	–	–
3	13 (13)	–	–
4	5 (5)	–	–
5	2 (2)	–	–
MDAS-question 2	35 (35)	0.203 (0.091–0.315)	< 0.001
1	4 (4)	–	–
2	5 (5)	–	–
3	9 (9)	–	–
4	7 (7)	–	–
5	10 (10)	–	–
MDAS-question 3	41 (41)	0.230 (0.112–0.348)	< 0.001
1	0 (0)	–	–
2	2 (2)	–	–
3	16 (16)	–	–
4	10 (10)	–	–
5	13 (13)	–	–
MDAS-question 4	35 (35)	0.192 (0.084–0.300)	< 0.001
1	2 (2)	–	–
2	5 (5)		–
3	14 (14)	–	–
4	9 (9)	–	–
5	5 (5)		–
MDAS-question 5	57 (57)	0.425 (0.300–0.550)	< 0.001
1	1 (1)	–	–
2	3 (3)	–	–
3	20 (20)	–	–
4	16 (16)	–	–
5	17 (17)	–	–

*CI* Confidence interval; *MDAS* Modified version of dental anxiety scale

Additionally, the kappa consistency test found that the consistency of children’s self-reported and mothers’ proxy-reported dental anxiety was highest in the third hierarchy, "fairly anxious", followed by the second hierarchy, "slightly anxious" (kappa coefficient = 0.028, *p* = 0.593). Notably, the kappa coefficient of the above consistency was extremely low and had no statistical significance. There was no agreement between the two groups in terms of anxiety hierarchies; that is, the mothers’ proxy ratings of children’s dental anxiety did not truly reflect the children’s self-reported dental anxiety (Table 5).

**Table 5:** Consistency analysis of children's self-reported and their mothers' proxy-reported dental anxiety hierarchies

Parameters	Consistency, n (%)	Kappa coefficient (95% CI)	*p* value
All hierarchies (degrees of anxiety)	42 (42)	0.028 (− 0.080–0.056)	0.593
No anxious (5–9 scores)	0 (0)	–	–
Slightly anxious (10–14 scores)	5 (5)	–	–
Fairly anxious (15–19 scores)	36 (36)	–	–
Very anxious (20–24 scores)	1 (1)	–	–
Extremely anxious (25 scores)	0 (0)	–	–

*CI* Confidence interval

### The factors affecting children’s dental anxiety in the unadjusted univariate model

A total of seven factors were involved in the univariate model for analysis, including age, gender, maternal anxiety, number of dental visits, mother’s presence or absence, oral health status, and having siblings or not (Table 6). In the univariate model, age [every 1-year increase, OR = 0.661, 95% confidence interval (CI) = 0.514–0.850, *p* = 0.001] and several dental visits (every 1-visit increase, OR = 0.409, 95%, CI = 0.190–0.880, *p* = 0.022) were factors affecting children’s dental anxiety. Moreover, the mother’s presence was associated with a 0.286-fold (95% CI = 0.114–0.714, *p* = 0.007) decrease in children’s dental anxiety risk compared with the mother's absence during dental treatment. However, other factors, such as gender, maternal anxiety, oral health status, and having siblings, were all excluded from the univariate model.

**Table 6:** Unadjusted univariate logistic regression analysis of the factors affecting children’s dental anxiety

Variable	No DA (n = 70)	DA (n = 30)	UOR (95% CI)	B	*p* value
Age (by every 1 year increase)	9 (7–11)	7 (6–8.3)	0.661 (0.514–0.850)	− 0.414	0.001
Gender, female	26 (37.1)	12 (40.0)	1.0 (Reference)	–	–
Gender, male	44 (62.9)	18 (60.0)	0.886 (0.369–2.130)	− 0.121	0.787
Without maternal anxiety	44 (62.9)	21 (70.0)	1.0 (Reference)	–	–
With maternal anxiety	26 (37.1)	9 (30.0)	0.725 (0.289–1.818)	− 0.321	0.493
Number of dental visits (by every 1 visit increase)	0.7 ± 0.9	0.2 ± 0.5	0.409 (0.190–0.880)	− 0.893	0.022
Mother absence	28 (40.0)	21 (70.0)	1.0 (Reference)	–	–
Mother presence	42 (60.0)	9 (30.0)	0.286 (0.114–0.714)	− 1.253	0.007
Not good oral health	39 (55.7)	19 (63.3)	1.0 (Reference)	–	–
Good oral health	31 (44.3)	11 (36.8)	0.728 (0.302–1.755)	− 0.317	0.480
Not having sibling	39 (55.7)	17 (56.7)	1.0 (Reference)	–	–
Having sibling	31 (44.3)	13 (43.3)	0.962 (0.406–2.279)	− 0.039	0.930

Data are presented as the median (interquartile range), mean ± standard deviation, or n (%). B, regression coefficient; *CI* Confidence interval; *DA* Dental anxiety; *UOR* Unadjusted odds ratios

### The factors affecting children’s dental anxiety in the adjusted multivariate model

Age, number of dental visits, and mother presence were further included in the multivariate logistic regression analysis (Table 7). Finally, after adjustments for possible confounders, i.e., the number of dental visits, age (every 1 year increase), and mother presence were associated with 0.697-fold (95% CI = 0.535–0.908, *p* = 0.007) and 0.362-fold (95% CI = 0.135–0.967, *p* = 0.043) decreases in the risk of children’s dental anxiety during dental treatment in the multivariate model.

**Table 7:** Adjusted multivariate logistic regression analysis of the factors affecting children’s dental anxiety

Variable	No DA (n = 70)	DA (n = 30)	AOR (95% CI)	B	*p* value
Age (by every 1 year increase)	9 (7–11)	7 (6–8.3)	0.697 (0.535–0.908)	− 0.361	0.007
Number of dental visits (by every 1 visit increase)	0.7 ± 0.9	0.2 ± 0.5	0.486 (0.212–1.111)	− 0722	0.087
Mother absence	28 (40.0)	21 (70.0)	1.0 (Reference)	–	–
Mother presence	42 (60.0)	9 (30.0)	0.362 (0.135–0.967)	− 1.017	0.043

Data are presented as the median (interquartile range), mean ± standard deviation, or n (%). B, regression coefficient; *CI* Confidence interval; *DA* Dental anxiety; *AOR* Adjusted odds ratios

## Discussion

Dental anxiety is common among children and adults seeking treatment in dental clinics. In the current study, a new scale was developed by combining the MDAS and FIS (MDAS-FIS). Additionally, the current study found that children’s self-reported dental anxiety is commonly underestimated by their mothers’ proxy-reported dental anxiety during dental visits, and with increasing age, the degree of dental anxiety in children gradually decreases, and the presence of mothers during dental visits can alleviate dental anxiety in children.

In dental clinics, timely detection and appropriate intervention can shorten the time and frequency of seeking treatment and help relieve the economic and psychological burden of patients [4, 7, 12–16]. As early as 1968, Corah et al. designed a questionnaire to evaluate the levels of dental anxiety, namely, the Corah Version of the Dental Anxiety Scale (CDAS) [17, 18]. Four questions were designed for the CDAS, while five questions were designed for the MDAS in 1995 [9]. The first four questions of the two scales were consistent (Table 1) [9]. The fifth problem of MDAS involves local anesthesia injection because it is a common concern and even anxiety among patients attending the department of stomatology [9]. Both the CDAS and MDAS were initially used for the assessment of dental anxiety in adults, and then a large number of studies gradually began to apply them to the study of dental anxiety in children. Many studies have found that MDAS has good predictive effects in both adults and children [5, 6].

The FIS includes five images, ranging from very sad (very worried) to very happy (relaxed or not worried) [10]. FIS is used to detect the anxiety state of children attending hospitals and can be used as a single detection method in clinical studies [10, 19–22]. In addition, several studies have used FIS to describe the anxiety state of MCDAS and CDAS, i.e., from 1 to 5 representing no anxiety to very anxiety of MCDAS and CDAS, respectively [23–27]. Therefore, we combined the MDAS with the FIS to detect dental anxiety in both children and their mothers in the current study.

Previous studies have suggested that children aged 8 and above can accurately express their physical and mental discomfort [28]. However, many studies have found that children aged 3–4 can communicate physical and mental experiences, such as pain. [29, 30]. The children included in this study were all primary school students in Zhengzhou City, with a minimum age of 6 years, a maximum age of 12 years, and a median age of 8.5 (7.0–10.8) years, which met the age requirements of previous studies.

Although there are many international studies on dental anxiety in children [2, 5, 6], there are relatively few studies on the difference between children’s self-reported and parents’ proxy-reported dental anxiety [1, 8, 26]. Limited studies suggest that the reliability of parents’ proxy-reported dental anxiety is poor and cannot replace children's self-reported dental anxiety; that is, the consistency between the two is poor [6, 31, 32]. In 2015, a study found that parents’ proxy-reported dental anxiety underestimated 46% of children's self-reported dental anxiety in the UK [1]; the results of this study suggest that more attention should be given to the screening of children's self-reported dental anxiety. In addition, from a macro perspective, children's self-reporting of a clinical problem is conducive to a more accurate assessment of the incidence, risk factors and clinical manifestations of the clinical problem in children to facilitate subsequent prevention and treatment strategies [6].

In this study, it was found that MDAS-Question 5 was the item with the highest consistency of children’s self-reported and mothers’ proxy-reported dental anxiety, regardless of the scores and hierarchies (degrees or stratifications), but only moderate consistency, followed by MDAS-Question 3, with the only general agreement (Table 4). MDAS-Question 1 was the item with the lowest consistency (Table 4). The results of this study suggest that even on MDAS-Question 5 with the best consistency, mothers’ proxy-reported dental anxiety underestimates children’s self-reported dental anxiety by 43% (Table 4). In terms of dental anxiety stratification (hierarchy), the consistency was worse, and there was no significant difference (Table 5). This study highlights the importance of early detection and early intervention of children’s self-reported dental anxiety.

The reason for the difference between children’s self-reported and mothers' proxy-reported dental anxiety is unclear. Our clinical observations suggest that this may be related to the cognitive differences between children and their mothers in dental diagnosis and treatment or may be due to their mothers' overoptimistic assessment of their children's dental diagnosis and treatment performance. In addition, it is not known whether the differences between children's self-reported and their mothers' proxy-reported dental anxiety gradually decrease as children age. If the answer is yes, it may be understood that the difference between children's self-reported and parents’ proxy-reported dental anxiety is due to the children's low awareness of dental care, since parental awareness generally does not fluctuate greatly.

Notably, the current study has originality and significance compared with previous studies [1, 8, 26]. First, the ages of the study children were the youngest, and the median or mean ages of the children in the other three studies were approximately 10 years or above. Second, the measurement scales are different from each other. Our study employed a sample and a suitable scale, i.e., MDAS-FIS, and the other three studies employed the Children’s Fear Survey Schedule (15 items) and Modified Child Dental Anxiety Scale-faces version (6 items). Third, we not only checked the interrater agreement of the items but also tested the interrater agreement of the hierarchies (degrees or stratifications); the other studies only performed one of them. Fourth, we investigated the factors affecting children’s dental anxiety in the same cohort of children simultaneously, and other studies did not perform this investigation. Regardless of the differences, our study combined with previous studies has a similar finding that the interrater agreement between children’s self-reported and their mothers’ proxy-reported dental anxiety is poor, which indicates that children’s self-reported dental anxiety is relevant and that mothers’ proxy-reported dental anxiety is an important supplement.

However, there are some limitations in this study. First, family socioeconomic status was not investigated. Different socioeconomic conditions could lead to various understandings of MDAS questions in children and their mothers. Notably, all participants were urban primary school students, and all their parents had decent jobs in Zhengzhou City. The favorable cognitive ability and representativeness of the participants can largely reduce the potential bias of the socioeconomic conditions. Second, we only enrolled mothers rather than the fathers of children in this study because we found that mothers were more approachable to their children during the elementary study period, and more than 90% of accompanying dental visits were performed by only mothers in real-life dental practice. Therefore, we do not know whether the findings would be different if the mothers were replaced by the fathers. Third, many patients were excluded from the study according to the study design, and the effect on the findings of the study is unknown; however, the finally enrolled participants were more homogeneous to draw a valid conclusion. Last, given that the sample size of this study is small, we did not score dental anxiety as a scale but on a continuum and performed linear regressions rather than logistic regressions; future large sample size studies using linear regression are warranted.

In conclusion, the prevalence of children’s self-reported dental anxiety is generally underestimated by their mothers; children should be encouraged to test their dental anxiety by scales (such as the MDAS-FIS) by themselves before dental diagnosis and treatment to facilitate early detection and intervention. Additionally, maternal presence during dental visits is strongly recommended for children.

## Data Availability

All data generated or analyzed during this study are included in this published article.
